# Supplementary material for: Rapid turnover of life-cycle-related genes in the brown algae
Source: Genome Biol. 2019 Feb 14;20:35. doi: 10.1186/s13059-019-1630-6 (PMC6374913; doi:10.1186/s13059-019-1630-6)
Supplement: Supplementary file 3 — Protein alignments, corresponding Gblocks html file and CDS sequences for the lineage conserved gametophyte-biased genes. (ZIP 2109 kb) [file 13059_2019_1630_MOESM3_ESM.zip › Con_GA_alignments/Ec-11_006190.aa_ali.fasta-gb.htm]

Ec-11\_006190.aa.fasta.trans.aa\_ali.fasta


## Gblocks 0.91b Results

Processed file: **Ec-11\_006190.aa.fasta.trans.aa\_ali.fasta**  
Number of sequences: **2**  
Alignment assumed to be: **Protein**  
New number of positions: **470** (selected positions are underlined in blue)

```
                         10        20        30        40        50        60
                 =========+=========+=========+=========+=========+=========+
Ec-11_006190     MGASPLRTRIRPIKDQDVQVAQESPPPPTFTVPAFRPRKHRGGESSLLKDAYPSGFCRPP
MSTRG.59175.1    ------------------------------------------------------------
                                                                             


                         70        80        90       100       110       120
                 =========+=========+=========+=========+=========+=========+
Ec-11_006190     QNRPLPATTTTTATDTRSTRRKMVWLSGPPRQDLLHESKHVGVPPCGVASSRRILKSTRV
MSTRG.59175.1    ------------------------------------------------------------
                                                                             


                        130       140       150       160       170       180
                 =========+=========+=========+=========+=========+=========+
Ec-11_006190     VIGAPDVVDTVGSELVQFGPGFSIGPAEHYSPRRCTLTPDRPRPRTPTTPISPSRMTIDG
MSTRG.59175.1    --------XTAG--LQSLSP----------TGVQCLTSEGR-----------------EG
                                                                            #


                        190       200       210       220       230       240
                 =========+=========+=========+=========+=========+=========+
Ec-11_006190     STLLGRSSLLVEPRAVDRQNQRPGLKIRPPSEDNIYHMYDMEGHKGVRPTTADRNQASAG
MSTRG.59175.1    SMGLNVSDEADEGQAYDR----------------------------VFGLKEEITAAAIG
                 ##################                                          


                        250       260       270       280       290       300
                 =========+=========+=========+=========+=========+=========+
Ec-11_006190     PEPWPFPCDKLTERSSRPGTTAQVQETASWNRGDPGDDVFAAPHASDCGHDGRTLGRDDE
MSTRG.59175.1    ---------------TGGGATRQQTDTVCQS--------AQSPHHDDS----------DT
                                                                           ##


                        310       320       330       340       350       360
                 =========+=========+=========+=========+=========+=========+
Ec-11_006190     AETAAAGADQQTQSASQLSTPRVHHVDNENNTSEHGAVNTPDARGQTPTEKPKLRIEDCR
MSTRG.59175.1    PSDPCAVKDKNDRTPSVMSKIEVALDSP--------GAKVPKVRNQEERE------LDVR
                 #######################                 ##########       ###


                        370       380       390       400       410       420
                 =========+=========+=========+=========+=========+=========+
Ec-11_006190     NERVGRSQEEVELGNEDELLDGGKTGEAWAVDGKDDPSVWSGDRSGGILASVTTNGNSAV
MSTRG.59175.1    DEKSNGSLG-TRVLDEDLLSDGVRVEEAWAIDNKDNPSVWS-CCSAHILASVATNNSSAV
                 #######        ##########################   ################


                        430       440       450       460       470       480
                 =========+=========+=========+=========+=========+=========+
Ec-11_006190     CSSSIRDDISVEHVSAQRSSSTLDTGLYRGILSKDATDILRFHLESKNKPSGNAEACFSA
MSTRG.59175.1    FNSSVGEG-SVEPLSAKRSFSTLDAERYRESLSRDANEILRSHLKSRNEPSSETNSIFSN
                 ####     ###################################################


                        490       500       510       520       530       540
                 =========+=========+=========+=========+=========+=========+
Ec-11_006190     SCDALAGDTSSLI---------SQLLDNPLAARDREYSALFRLPSIDRSPEMCFVRTKSD
MSTRG.59175.1    SRQVVTSAATAVLSPAPPPARQRQLLDDLLAARGQEHPPQFRLDPMERSPEMCYVRTRSD
                 #                      #####################################


                        550       560       570       580       590       600
                 =========+=========+=========+=========+=========+=========+
Ec-11_006190     VILAVVRKKELLECDDDAGLRNLKTAACPVRIQADMTLAEGMREISASGALRHAPNKE--
MSTRG.59175.1    LVLAVVRKEKLLDCADDGSFRSLKTAARPVRIKADMDLADGLRVILASGVLRDDATKVDI
                 #########################################################   


                        610       620       630       640       650       660
                 =========+=========+=========+=========+=========+=========+
Ec-11_006190     ------SR---WGGSRIG--RAGVKGDTSG-AGRTQTLEYFNSQAGAWEVVRNETDWKVL
MSTRG.59175.1    GLLIERSRSHVSGGRSVAGAEAGAGNDIQGTSGQAQALEYFNPYAGTWETVRNESDWRVF
                                                 ############################


                        670       680       690       700       710       720
                 =========+=========+=========+=========+=========+=========+
Ec-11_006190     LAAPAGPQGAGAVFQS--AGENDRRADFLVAVS---PTPTLKGTRSSNDNTASVRPGPDL
MSTRG.59175.1    LTAQAGPQGAGVVFQSTRAGASVVRADFLVAVTSTRPPVSLEMTRSSKSRAPSRYAWSNA
                 ################  ##############    #################       


                        730       740       750       760       770       780
                 =========+=========+=========+=========+=========+=========+
Ec-11_006190     DPGSKA----SR---SGWRYSSSVTPGTLRQVRASGHSQDPVLSDTHEAGTEAESHQEGE
MSTRG.59175.1    TRQPHAARRVLREKPLDWRYATAVTPGVLRSSKDSNGERNESQ-DTTNTQAMAEDVRRGV
                                  ##################         ################


                        790       800       810       820       830       840
                 =========+=========+=========+=========+=========+=========+
Ec-11_006190     DDNFRRRLVALAGGAKRAGPGPGAALWLPASTRTSAAVGRGTAAASPTHRPNKAKVRRLV
MSTRG.59175.1    RDDFRQRLADLDGETRIAGPGPGAALWMTTSTRPHNFCPKATANSSP-HRPRKDKVRRLV
                 ############################################### ############


                        850       860       870       880       890
                 =========+=========+=========+=========+=========+===
Ec-11_006190     RHLEAGLKLKGRPGAGGLPVGSQKIDMPYTKELAKWSYDEILGIPRTAGP--T
MSTRG.59175.1    RHLEAGLMLKGQPGAGGLPLGGQKIDMPYTKELAKWSYDEILGIPRATATASA
                 ##############################################
```

```
Parameters used
Minimum Number Of Sequences For A Conserved Position: 2
Minimum Number Of Sequences For A Flanking Position: 2
Maximum Number Of Contiguous Nonconserved Positions: 8
Minimum Length Of A Block: 10
Allowed Gap Positions: None
Use Similarity Matrices: Yes
```

```
Flank positions of the 14 selected block(s)
Flanks: [180  198]  [299  323]  [341  350]  [358  367]  [376  401]  [405  424]  [430  481]  [504  597]  [633  676]  [679  692]  [697  713]  [738  755]  [765  827]  [829  886]  

New number of positions in Ec-11_006190.aa.fasta.trans.aa_ali.fasta-gb:  470  (52% of the original 893 positions)
```
